# Supplementary material for: Identification of Freezing-Responsive microRNAs and Their Targets in Chinese Jujube by Small RNA and Degradome Sequencing
Source: Int J Mol Sci. 2026 Sep 4;27(17):7912. doi: 10.3390/ijms27177912 (PMC13566890; doi:10.3390/ijms27177912)
Supplement: Supplementary file 1 [file ijms-27-07912-s001.zip › Supplementary Material S2/novel_miR_125.pdf]

Provisional ID	:	novel_miR_125
Score total	:	302.1
Score for star read(s)	:	3.9
Score for read counts	:	300.4
Score for mfe	:	0
Score for randfold	:	-2.2
Score for cons. seed	:	
Total read count	:	601
Mature read count	:	272
Loop read count	:	328
Star read count	:	1

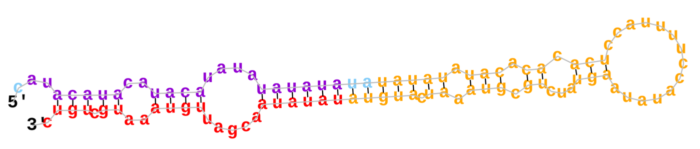

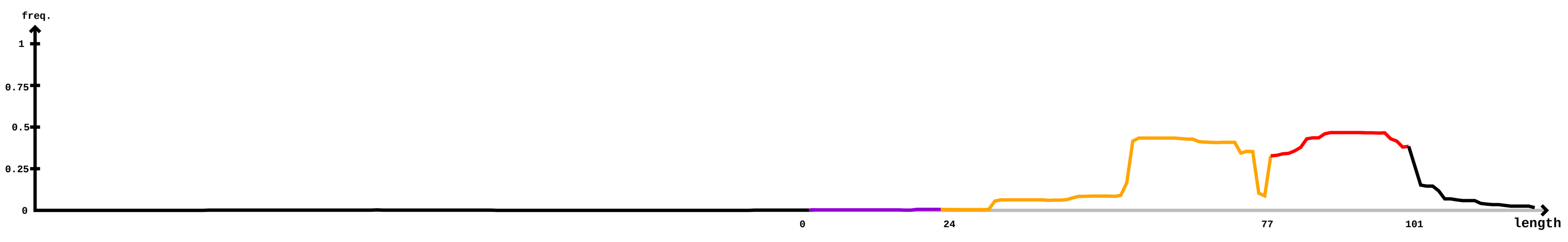

Star **Mature**

```
5'- uucuuaagagagaucaaauugauaaaaccaucugaccgguagacauucaaaagaguauguuagaaucaugcucaaagauuugcacgaaauuaagggagcaggaaauacauacaugcaaacauacauacauacauacauacauauauauauauauauauauacacacacuccauuuuccauauaaguaucugcguaaaucauguauauauaacgauuguaaaugcuguccauggauguagacuguaaggac -3'  obs
    uucuuaagagagaucaaauugauaaaaccaucugaccgguagacauucaaaagaguauguuagaaucaugcucaaagauuugcacgaaauuaagggagcaggaaauacauacaugcaaacauacauacauacauacauacauauauauauauauauauauacacacacuccauuuuccauauaaguaucugcguaaaucauguauauauaacgauuguaaaugcuguccauggauguagacuguaaggac       exp
    .((((....)))).................(((...((((..(((((((.....((((((.....((.(((((...(((((.....)))))....))))).))...))))))..............(((..(((...((((....((((((((((((.(((.((.(((..............)))...)).))).)).)))))))))).....)))).))).)))...)))))))..))))...))).    reads  mm  sample
    ...........................ccaucugaccgguagacauucaaaagagu..................................................................................................................................................................................................    1      0   JCK-2
    .......................................................uauguuagaaucaugcucaa...............................................................................................................................................................................    1      0   LT-3
    ......................................................................................................................acauacauacaCacauacauacaua...........................................................................................................    1      1   LT-1
    ................................................................................................................................auacauacauacaCauauauaua...................................................................................................    1      1   LT-2
    .................................................................................................................................................uaCauauauauauauacacaca...................................................................................    3      1   LT-2
    .............................................................................................................................................................uauaUacacacuccauuuucc........................................................................    1      1   JCK-1
    ..............................................................................................................................................................auaUacacacuccauuuucca.......................................................................    1      1   JCK-1
    ..............................................................................................................................................................auaUacacacuccauuuucca.......................................................................    4      1   LT-3
    ..............................................................................................................................................................auaUacacacuccauuuucca.......................................................................    8      1   LT-2
    ..............................................................................................................................................................auaUacacacuccauuuucca.......................................................................    17     1   LCK-3
    ..............................................................................................................................................................auaUacacacuccauuuucca.......................................................................    1      1   LT-1
    ..............................................................................................................................................................auaUacacacuccauuuuccau......................................................................    2      1   LT-2
    ...............................................................................................................................................................uaUacacacuccauuuuccau......................................................................    1      1   LT-1
    ...............................................................................................................................................................uaUacacacuccauuuuccau......................................................................    3      1   LT-3
    ...............................................................................................................................................................uaUacacacuccauuuuccaua.....................................................................    1      1   LT-1
    .......................................................................................................................................................................cuccauuuuccauauaaguauc.............................................................    1      0   LT-1
    ........................................................................................................................................................................uccauuuuccauauaaguauc.............................................................    1      0   LT-1
    ..........................................................................................................................................................................cauuuuccauauaaguaucu............................................................    2      0   LT-3
    ...........................................................................................................................................................................auuuuccauauaaguaucugc..........................................................    3      0   LT-3
    ...........................................................................................................................................................................auuuuccauauaaguaucugc..........................................................    2      0   JCK-2
    ...........................................................................................................................................................................auuuuccauauaaguaucugc..........................................................    2      0   LT-1
    ............................................................................................................................................................................uuuuccauauaaguaucugc..........................................................    3      0   LT-2
    ............................................................................................................................................................................uuuuccauauaaguaucugcg.........................................................    2      0   LT-3
    ............................................................................................................................................................................uuuuccauauaaguaucugcgu........................................................    1      0   LT-1
    ..............................................................................................................................................................................uuccauauaaguaucugcgua.......................................................    1      0   LT-1
    ...................................................................................................................................................................................uauaaguCucugcguaaauc...................................................    1      1   LCK-3
    ...................................................................................................................................................................................uauaaguaucugcguaaauc...................................................    1      0   JCK-1
    ...................................................................................................................................................................................uauaaguaucugcguaaauc...................................................    7      0   LCK-3
    ...................................................................................................................................................................................uauaaguaucugcguaaauc...................................................    7      0   LT-2
    ...................................................................................................................................................................................uauaaguaucugcguaaauc...................................................    2      0   JCK-2
    ...................................................................................................................................................................................uauaaguaucugcguaaauc...................................................    6      0   LT-3
    ...................................................................................................................................................................................uauaaguaucugcguaaauc...................................................    3      0   LT-1
    ...................................................................................................................................................................................uauaaguaucugcguaaauc...................................................    3      0   JCK-3
```

Star Mature

uucuuaagagagaucaaauugauaaaaccaucugaccgguagacauucaaaagaguauguuagaaucaugcucaaagauuugcacgaaauuaagggagcaggaaauacauacaugcaaacauacauacauacauacauacauauauauauauauauauauacacacacuccauuuuccauauaaguaucugcguaaaucauguauauauaacgauuguaaaugcuguccauggauguagacuguaaggac

uauaaguaucugcguaaauca	1	0	LT-3
uauaaguaucugcguaaauca	2	0	LT-1
uauaaguaucugcguaaauca	1	0	JCK-3
uauaaguaucugcguaaauca	1	0	JCK-2
auaaguaucugcguaaaucaug	1	0	LT-1
auaaguaucugcguaaaucaug	9	0	LT-3
auaaguaucugcguaaaucaug	11	0	LCK-3
auaaguaucugcguaaaucaug	8	0	JCK-2
auaaguaucugcguaaaucaug	3	0	JCK-1
auaaguaucugcguaaaucaug	23	0	LT-2
auaaguaucugcguaaaucaug	1	0	JCK-3
uaaguaucugcguaaauc	13	0	LCK-3
uaaguaucugcguaaaucau	1	0	JCK-1
uaaguaucuAcguaaaucaug	1	1	JCK-3
uaaguaucugcguaaaucaug	21	0	LT-2
uaaguaucugcguaaaucaug	23	0	LCK-3
uaaguaucugcguaaaucaug	35	0	JCK-2
uaaguaucugcguaaaucaug	16	0	JCK-3
uaaguaucugcguGaaucaug	1	1	JCK-2
uaaguaucugcguaaaucaug	14	0	JCK-1
uaaguaucugcguaaaucaug	18	0	LT-1
uaaguaucugcguaaaucaug	23	0	LT-3
uaaguaucugcguaaaucaugu	2	0	LT-2
aaguaucugcguaaaucaugu	3	0	LT-3
aCguaucugcguaaaucaugu	1	1	LT-3
aaguaucugcguaaaucaugu	6	0	LCK-3
aaguaucugcguaaaucaugu	1	0	LT-2
aaguaucugcguaaaucaugua	1	0	LT-1
aucauguauauauaacgauuguaa	1	0	LT-3
uguauauauaacgauuguaaau	1	0	LT-3
uguauauauaacgauuguaaaug	1	0	LT-3
uguauauauaacgauuguaaaugc	1	0	JCK-2
uguauauauaacgauuguaaaugc	2	0	JCK-1
uguauauauaacgauuguaaaugc	1	0	JCK-3
uguauauauaacgauuguaaaugc	2	0	LT-2
uguauauauaacgauuguaaaugc	3	0	LCK-3
uguauauauaacgauuguaaaugcu	1	0	LT-2
uauauauaacgauuguaaaugc	2	0	JT-3
uauauauaacgauuguaaaugc	3	0	LT-3
uauauauaacgauuguaaaugc	1	0	LT-2
uaNauauaacgauuguaaaugc	1	1	LT-2
uauauauaacgauuguaaaugc	3	0	LT-1
uauauauaacgauuguaaaugc	2	0	JCK-1
uauauauaacgauuguaaaugc	3	0	JCK-2
uauauauaacgauuguaaaugcug	6	0	LCK-3
uauauauaacgauuguaaaugcug	1	0	LT-2
uauauauaacgauuguaaaugcug	5	0	JCK-1
uauauauaacgauuguaaaugcug	8	0	JCK-2
uauauauaacgauuguaaaugcug	5	0	JCK-3
uauauauaacgauuguaaaugcuguc	2	0	JCK-2
auauauaacgauuguaaaug	1	0	LT-3
auauauaacgauuguaaaugcuguc	1	0	LT-2
uauauaacgauuguaaaugcu	1	0	LT-3
uauauaacgauuguaaaugcu	7	0	LCK-3
uauauaacgauuguaaaugcuguc	3	0	JT-2
uauauaacgauuguCaaugcuguc	1	1	JCK-2
uauauaacgauuguaaaugcuguc	3	0	LT-2
uauauaacgauuguaaaugcuguc	12	0	LCK-3
uauauaacgauuguaaaugcuguc	13	0	LT-1
uauauaacgauuguaaaugcuguc	35	0	JCK-3
uauauaacgauuguaaaugcuguc	12	0	LT-3
uauauaacgauuguaaaugcuguc	35	0	JCK-1
uauauaacgauuguaaaugcuguc	4	0	JT-3
uauauaacgauuguaaaugcuguc	34	0	JCK-2
uCuauaacgauuguaaaugcuguc	1	1	LT-3
auauaacgauuguaaaugcuguc	2	0	JCK-2
uauaacgauuguaaaugcuguc	1	0	JCK-2
uauaacgauuguaaaugcuguc	1	0	LT-3
uauaacgauuguaaaugcuguc	2	0	JCK-1
uauaacgauuguaaaugcuguc	1	0	LT-1

```
                                                                                                                                                  Star                                                                     Mature
uucuuaagagagaucaaauugauaaaaccaucugaccgguagacauucaaaagaguauguuagaaucaugcucaaagauuugcacgaaauuaagggagcaggaaauacauacaugcaaacauacauacauacauacauacauauauauauauauauauauacacacacuccauuuuccauauaaguaucugcguaaaucauguauauauaacgauuguaaaugcuguccauggauguagacuguaaggac
..............................................................................................................................................................................................................uauaacgauuguaaaugcugucca....................    1    0    JCK-3
...............................................................................................................................................................................................................auaacgauuguaaaugcuguc......................    2    0    LT-1
................................................................................................................................................................................................................uaacgauuguaaaugcugucca....................    2    0    LT-1
................................................................................................................................................................................................................uaacgauuguaaaugcuguccaug..................    2    0    LT-2
................................................................................................................................................................................................................uaacgauuguaaaugcuguccaug..................    3    0    JCK-3
................................................................................................................................................................................................................uaacgauuguaaaugcuguccaug..................    2    0    JCK-2
................................................................................................................................................................................................................uaacgauuguaaaugcuguccaug..................    1    0    LT-1
.................................................................................................................................................................................................................aacgauuguaaaugcuguc......................    2    0    LT-3
.................................................................................................................................................................................................................aacgauuguaaaugcuguccaug..................    2    0    LT-2
.................................................................................................................................................................................................................aacgauuguaaaugcuguccaug..................    2    0    LT-3
.................................................................................................................................................................................................................aacgauuguaaaugcuguccaugg.................    1    0    LT-1
.................................................................................................................................................................................................................aacgauuguaaaugcuguccaugg.................    1    0    JCK-1
.................................................................................................................................................................................................................aacgauuguaaaugcuguccaugg.................    3    0    LT-2
.................................................................................................................................................................................................................aacgauuguaaaugcuguccaugg.................    3    0    JCK-3
..................................................................................................................................................................................................................acgauuguaaaugcugucca....................    2    0    LT-3
..................................................................................................................................................................................................................acgauuguaaaugcuguccaug..................    2    0    LT-1
..................................................................................................................................................................................................................acgauuguaaaugcuguccaug..................    2    0    LT-3
..................................................................................................................................................................................................................acgauuguaaaugcuguccaug..................    1    0    JCK-1
..................................................................................................................................................................................................................acgauuguaaaugcuguccaugg.................    2    0    JCK-2
..................................................................................................................................................................................................................acgauuguaaaugcuguccaugg.................    1    0    JCK-1
..................................................................................................................................................................................................................acgauuguaaaugcuguccaugg.................    1    0    JCK-3
..................................................................................................................................................................................................................acgauuguaaaugcuguccaugg.................    2    0    LT-2
..................................................................................................................................................................................................................acgauuguaaaugcuguccaugg.................    2    0    LCK-3
..................................................................................................................................................................................................................acgauuguaaaugcuguccaugg.................    3    0    LT-1
..................................................................................................................................................................................................................acgauuguaaaugcuguccaugg.................    3    0    LT-3
..................................................................................................................................................................................................................acgauuguCaaugcuguccaugg.................    1    1    JT-1
..................................................................................................................................................................................................................acgauuguaaaugcuguccaugg.................    9    0    JT-1
..................................................................................................................................................................................................................acgauuguaaaugcuguccauggaG...............    1    1    LT-1
..................................................................................................................................................................................................................acgauuguaaaugcuguccauggau...............    1    0    LT-2
..................................................................................................................................................................................................................acgauuguaaaugcuguccauggCu...............    1    1    LT-2
...................................................................................................................................................................................................................cgauuguaaaugcuguccauA..................    1    1    LT-3
...................................................................................................................................................................................................................cgauuguaaaugcuguccauggaug..............    1    0    LT-2
...................................................................................................................................................................................................................cgauuguaaaugcuguccauggauguagac.........    2    0    JCK-1
.....................................................................................................................................................................................................................auuguaaaugcuguccaug..................    1    0    JCK-1
.....................................................................................................................................................................................................................auuguaaaugcuguccauggau...............    1    0    LT-1
.....................................................................................................................................................................................................................auuguaaaugcuguccauggauguag...........    1    0    LT-1
.....................................................................................................................................................................................................................auuguaaaugcuguccauggauguag...........    4    0    JCK-2
.....................................................................................................................................................................................................................auuguaaaugcuguccauggauguag...........    2    0    JCK-1
.....................................................................................................................................................................................................................auuguaaaugcuguccauggauguag...........    2    0    JCK-3
.....................................................................................................................................................................................................................auuguaaaugcuguccauggauguaga..........    2    0    JCK-3
.....................................................................................................................................................................................................................auuguaaaugcuguccauggauguagacug.......    1    0    JCK-3
.....................................................................................................................................................................................................................auuguaaaugcuguccauggauguagacug.......    2    0    JCK-2
......................................................................................................................................................................................................................uuguaaaugcuguccauggaug..............    2    0    LT-1
......................................................................................................................................................................................................................uuguaaaugcuguccauggauguag...........    1    0    JCK-3
......................................................................................................................................................................................................................uuguaaaugcuguccauggauguag...........    1    0    JCK-2
......................................................................................................................................................................................................................uuguaaaugcuguccauggauguaga..........    1    0    LT-3
...............................................................................................................................................................................................................................cuguccauggauguagacugu......    1    0    JCK-3
...............................................................................................................................................................................................................................cuguccauggauguagacugu......    2    0    JCK-1
..................................................................................................................................................................................................................................uccauggauguagacuguaagg..    1    0    LT-3
...................................................................................................................................................................................................................................ccauggauguagacuguaagg..    1    0    JCK-2
...................................................................................................................................................................................................................................ccauggauguagacuguaagg..    2    0    JCK-1
....................................................................................................................................................................................................................................cauggauguagacuguaagga.    1    0    LT-3
....................................................................................................................................................................................................................................cauggauguagacuguaagga.    2    0    LT-1
....................................................................................................................................................................................................................................cauggauguagacuguaagga.    8    0    JCK-1
.....................................................................................................................................................................................................................................auggauguagacuguaagg..    1    0    LT-3
......................................................................................................................................................................................................................................uggauguagacuguaagg..    1    0    LT-3
```
